# Using Large Language Models for Advanced and Flexible Labelling of Protocol Deviations in Clinical Development

**DOI:** 10.1007/s43441-025-00785-z

**Published:** 2025-05-13

**Authors:** Min Zou, Leszek Popko, Michelle Gaudio

**Affiliations:** 1 F.Hoffmann-La Roche AG, 4070 Basel, Switzerland; 2 Hoffmann-La Roche Limited, 7070 Mississauga Road, Mississauga, ON L5N 5M8 Canada

**Keywords:** Quality assurance, Good clinical practice, Study management, Large language model, Protocol deviations

## Abstract

**Background:**

As described in ICH E3 Q&A R1 (International Council for Harmonisation. E3: Structure and content of clinical study reports—questions and answers (R1). 6 July 2012. Available from: https://database.ich.org/sites/default/files/E3_Q%26As_R1_Q%26As.pdf): “A protocol deviation (PD) is any change, divergence, or departure from the study design or procedures defined in the protocol”. A problematic area in human subject protection is the wide divergence among institutions, sponsors, investigators and IRBs regarding the definition of and the procedures for reviewing PDs. Despite industry initiatives like TransCelerate’s holistic approach [Galuchie et al. in Ther Innov Regul Sci 55:733–742, 2021], systematic trending and identification of impactful PDs remains limited. Traditional Natural Language Processing (NLP) methods are often cumbersome to implement, requiring extensive feature engineering and model tuning. However, the rise of Large Language Models (LLMs) has revolutionised text classification, enabling more accurate, nuanced, and context-aware solutions [Nguyen P. Test classification in the age of LLMs. 2024. Available from: https://blog.redsift.com/author/phong/]. An automated classification solution that enables efficient, flexible, and targeted PD classification is currently lacking.

**Methods:**

We developed a novel approach using a large language model (LLM), Meta Llama2 [Meta. Llama 2: Open source, free for research and commercial use. 2023. Available from: https://www.llama.com/llama2/] with a tailored prompt to classify free-text PDs from Roches’ PD management system. The model outputs were analysed to identify trends and assess risks across clinical programs, supporting human decision-making. This method offers a generalisable framework for developing prompts and integrating data to address similar challenges in clinical development.

**Result:**

This approach flagged over 80% of PDs potentially affecting disease progression assessment, enabling expert review. Compared to months of manual analysis, this automated method produced actionable insights in minutes. The solution also highlighted gaps in first-line controls, supporting process improvement and better accuracy in disease progression handling during trials.

## Background

Ensuring ethical and scientific quality of clinical trials and protecting the rights, safety and well-being of trial participants are the key objectives of the International Council for Harmonisation of Technical Requirements for Registration of Pharmaceuticals for Human Use (ICH) Good Clinical Practice (GCP). Investigators must follow approved protocols, and sponsors are responsible for monitoring compliance and identifying PDs [1]. GCP guidelines emphasise the importance of timely PD reporting and trending to maintain data integrity, patient safety, and regulatory compliance.

While the ICG E3 Q&A R1 [2] offers a definition of PDs, interpretations vary across stakeholders. TransCelerate’s guidance [3] clarifies that ‘important’ PDs are those likely to impact data reliability or subject safety and advocates risk-based identification in line with ICH E6 R2. PD reporting and trending are essential to maintaining the integrity, safety and regulatory compliance of clinical trials. Timely identification and reporting of these deviations allow sponsors/investigators to mitigate potential risks and ensure that study data remains reliable for analysis. Effective PD tracking supports regulatory compliance, risk mitigation, and the implementation of corrective and preventative actions (CAPAs). Despite this, PD management remains highly manual, inconsistent and prone to misclassification—especially given the volume of unstructured, non-standardised data. This hampers the detection of systemic study conduct issues.

Standardising PD classification is essential, yet its application remains inconsistent across companies. Unstructured descriptions, covering areas such as informed consent, inclusion/exclusion criteria, interventions and prohibited medications, contain valuable information that traditional methods struggle to process. Richard and Reddy [4] explored NLP techniques for automatically classifying unstructured PD descriptions, relying on Term-Frequency Inverse-Document-Frequency (TF-IDF) [5] for text representation and feature extraction followed by extensive model training on a curated dataset of 60,000 manually labeled PDs, a resource-intensive and time-consuming process.

Building on advances in Pretrained Language Models (PLMs), particularly LLMs, we propose a scalable prompt-based approach that largely utilises the knowledge stored in LLMs to classify PDs, eliminating the need for feature engineering or task-specific model training [6].

One of the key challenges in protecting critical clinical endpoints is the difficulty of manually reviewing vast amounts of clinical data to identify systemic issues in disease progression handling, issues that could impact study endpoints and patient safety across studies. Currently, the primary quality control measures for our clinical trials involve manual, siloed reviews. Disease progression is assessed by multiple business functions at different stages; however, these reviews are not holistic, nor is there a collaborative approach to integrate insights across functions. As each function conducts its review independently and at different time points, they may focus on distinct aspects of disease progression while overlooking other study controls or protocol requirements. This fragmented approach limits the integration of insights across functions, increasing the risk of undetected PDs that may compromise data integrity, impact interim analysis, study endpoints, and patient safety.

In our experiment, we address this challenge by:

1. Labelling PDs that directly affect the timeliness or accuracy of disease progression assessments using LLM, as discussed in section “Methods”.
2. Integrating labelled PDs with other relevant clinical insights and presenting them through intuitive and purpose-built visualisations to enable efficient expert investigation, as detailed in section “Results”.

## Methods

### Prerequisites

To ensure data security and privacy, all data were stored and processed in Roche’s secure environment. Additionally, llama2-70b-chat-v1 model [7] was hosted within Roche’s secured Amazon Web Services (AWS) [8] environment. Patient data were anonymised, in line with Roche Data Ethics Principles [9], and PDs were tracked centrally per standard operating procedures. All studies followed consistent endpoint definitions using RECIST 1.1 [10] criteria.

### Data

PD data used in this project originated from the Roche Protocol Deviation Management System and included structured attributes such as category, subcategory, dates, rational and unstructured free text descriptions. Despite efforts to standardise subcategories via the Protocol Deviation Assessment Plan (PDAP) [11], variability across studies remained high, complicating trending analysis. Additionally, PD reporting was influenced by differences in language, training and documentation practices among site representatives.

PD records typically include several key attributes for classification and analysis as shown in the Table 1.

**Table 1:** Key PD data attributes for classification

Attributes	Definition
Category	The type of deviation (e.g. non-compliance with patient criteria, medication or dosing errors)
Subcategory	Additional details that further specify the deviation category
Description	A detailed account of the deviation, including affected visits or study time points
Latest_Decision	The outcome regarding the patient’s continued participation in the study following the deviation
Latest_Rationale	Justification for the latest decision
Deviation_Number	A unique identifier is assigned to each deviation
Deviation_Status	Indicates whether the deviation was classified as important, with important deviations included in the Clinical Study Report
Date_Observed	The date when the deviation was first noticed
Date_Occurred	The actual date when the deviation took place
Date_Reported	The date when the deviation was officially reported
Earliest_Date_Confirmed_Important	The earliest date the deviation was confirmed as an important deviation

Example PD categories and subcategories are summarised in Table 2.

**Table 2:** Example PD categories and subcategories

Category	Subcategory
Procedural	Tumour assessment missed
Medication	Over-dosage received
Inclusion criteria	Age criteria not met
Exclusion criteria	Prohibited concurrent medication

### Prompt-Based Approach Using LLMs for Large-Scale Text Classification

**Figure 1:**
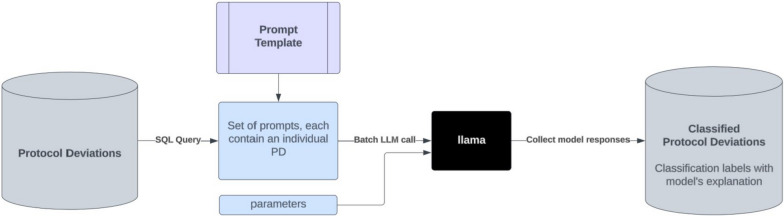
Schematic representation of the automated batch classification workflow

Rather than utilising LLMs for conversation or chat-based tasks, we adapted them for large-scale classification and labeling of unstructured text within clinical development databases. Our approach consists of the following key steps:

1. Prompt Strategy Development: We designed a prompt strategy tailored to our use case, considering key technical factors.
2. Prompt Template Development: Using a small sample set, we manually tested the model’s responses to develop and refine prompt templates and parameter configurations.
3. Batch Classification Process: An automated batch process, as illustrated in Fig. 1 was implemented to classify multiple PDs using the selected prompt template from Step 2 (represented by the purple box) and specified parameters. The outputs were stored as additional attributes in JSON format.
4. Prompt Evaluation: We assessed the performance of the prompts using a randomly selected, expert-curated dataset. The selected prompt templates from Step 2 were batch-processed in Step 3, and the aggregated results were evaluated in Step 4.

Steps 2, 3, and 4 may be repeated for iterative prompt refinement, with each step detailed in sections “Step 1: Prompt Strategy Development” to “Step 4: Prompt Evaluation”.

#### Step 1: Prompt Strategy Development

The prompt-based approach using LLMs starts with a well-defined prompt strategy. The task’s objective must be clearly identified, along with precise instructions for execution and expected output.

Several key technical factors were considered in developing the prompt strategy:

##### Context Window and Token Limits

LLMs do not process text as raw words or sentences but instead break input into tokens using a tokenization algorithm [12]. While LLMs can handle long contexts, their ability to process extensive text within a single prompt is constrained by the model’s context window, the maximum number of tokens the model can ‘remember’ during inference [13]. This limitation may affect decision-making tasks that require analysing large amounts of context. To optimise performance, prompt strategies should focus on concise, structured prompts that include only essential information while maintaining clarity and ensuring tasks can be completed with straightforward instructions.

##### Randomness and Diversity

Randomness and diversity [14] refer to the degree of variation in a model’s responses. In Llama 2, these can be adjusted using two specific parameters:

*Temperature*: Adjusts the probability distribution of predicted outputs. Lower values make responses more deterministic, while higher values increase randomness, allowing more exploratory outputs.

*Top P*: Dynamically restricts token selection based on cumulative probability mass. Lower values limit choices to high-confidence predictions, improving consistency, whereas higher values allow broader variability in outputs.

For text classification, if the goal is to minimise false positives and ensure strict adherence to defined criteria, a lower temperature and lower top-P setting may be preferable. Conversely, if the goal is to capture as many potential records as possible, as in our use case, even with some uncertainty, a higher Temperature and higher Top-P setting may be beneficial.

##### Zero-Shot versus Few-Shot

Zero-shot refers to the ability of a model to perform a task, such as PD classification, without requiring explicit examples for the task included in the prompt. The model relies on its pre-existing knowledge, such as language patterns and domain understanding, to infer the correct output based solely on the given prompt [15].

Few-shot, on the other hand, involves providing the model with a small number of examples within the prompt to guide its task execution. These examples are believed to help the model learn the context and recognise patterns relevant to the task, enhancing its ability to generalise to new instances [16]. The application of such techniques is detailed in sections “Step 2: Prompt Template Development” and “Step 4: Prompt Evaluation”.

##### Prompt Robustness

Minor changes in prompt phrasing may greatly affect model outputs [17–19]. Prompt strategies should account for this LLM behavior by testing multiple prompt variations across different records to ensure consistent and reliable classifications. Additionally, a fallback mechanism can be incorporated to flag uncertain cases for manual review. Before adopting prompts into production, additional performance metrics such as sensitivity and consistency [20] should be considered to select the best-performing prompt.

With these considerations in mind, we designed a prompt strategy specifically for our classification task. Our goal was to identify PDs that may impact disease progression based on specified criteria, prioritising recall over precision to ensure the capture of as many potential positive cases as possible for expert review (see sections “Step 4: Prompt Evaluation” and “Discussion” for further details). We aimed for a more flexible model behavior to better handle nuances and identify relevant cases. However, without prior knowledge of how different parameter settings would affect prompt performance, we started with low values, closer to a deterministic configuration and gradually increased the temperature and top_P. Furthermore, due to high variability in PD reporting and, in some cases, insufficient detail leaving room for interpretation, we defined a three-tiered classification schema that included a “MaybeDP” option to allow the model to flag uncertain cases as a fallback mechanism. Additionally, the model’s explanation was included as part of its response to enhance robustness and transparency:

- “DP”: Records related to assessing or handling disease progression, including investigator assessments, clinically assessed as disease progression or incorrect assessments.
- “Maybe DP”: Records suggest potential impacts on disease progression but lack sufficient detail for a definitive classification.
- “No DP”: Records unrelated to disease progression.

#### Step 2: Prompt Template Development

Guided by the defined prompt strategy, prompt template development begins with manually prompting the model using an initial template, structured as follows:

- System message: Defines the model’s role and task objective
- User message: Contains task instructions, classification criteria, expected response format and the actual record for classification.
- Model configuration parameters:
  - max_gen_len: Limits response length to 2048 tokens,
  - temperature: Set to 0.1 (low randomness, more deterministic output),
  - top_p: Set to 0.1 (restricts token selection to highly probable choices).

**Figure 2:**
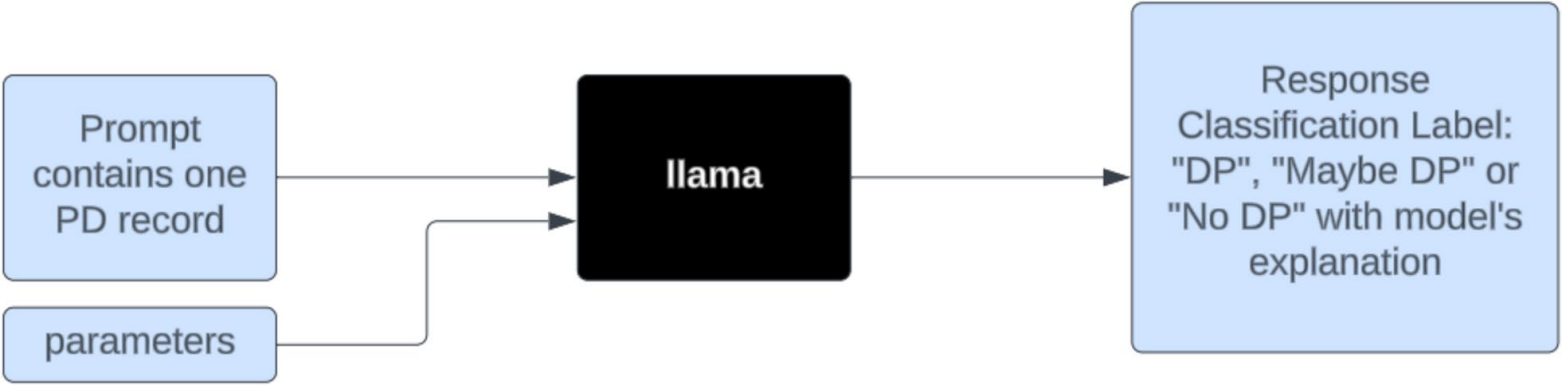
Schematic representation of manually prompting for PD classification, one record at a time

Figure 2 illustrates this manual process of handling PD classification one record at a time. This process is repeated many times to iteratively refine the prompt template, gradually increasing temperature and top_p while reviewing model responses for accuracy and consistency using a small sample of test records.

Prompt refinement included in this experiment are detailed in Table 3.

**Table 3:** Summary of the prompting techniques applied in the experiment

Short name	Description
Reword	Adjust phrasing for clarity
Pos	Provide the model with positive feedback before querying it. Some experiments suggested that giving positive reinforcement can lead to better performance on text classification tasks [21]
B-context	Provide business context for the task, e.g. the impact of delayed disease progression detection or a brief summary of RECIST 1.1 or explanation of labels used for response assessment per RECIST 1.1
C-context	Provide more context for the classification criteria
D-context	Provide more context for the record, e.g. definition of PD attributes
example	Add examples following few-shot techniques
nondialogue	Ask the model ignore prior conversation history and not generate dialogue
strict	Ask the model to base its decisions strictly on explicit facts, avoiding assumptions
heading	Add headings to structure each key section

Based on the manual test results, three significantly different prompt versions, as shown in Table 4, were selected for the batch classification process and further evaluation.

**Table 4:** Prompt versions selected for further evaluation

Prompt version	Adjustments
1	B-context + C-context + D-context + example
2	reword + pos + nondialogue + strict
3	reword + pos + B-context + D-context + example + heading

#### Step 3: Batch Classification Process

The prompt templates selected in section “Step 2: Prompt Template Development” are tested through an automated batch classification process, as shown in Fig. 1. The process starts with extracting PDs from a database via an SQL query [22]. These deviations are then formulated into a set of prompts, each containing an individual deviation and structured according to a predefined prompt template with relevant parameters. These prompts are fed into the LLM through a batch call, allowing the model to process multiple prompts automatically. The responses from the model are collected, classifying each record as “DP”, “Maybe DP”, or “No DP”, along with explanatory details. The classified deviations are then stored in a target database for further analysis or action.

Various parameter configurations and prompt versions are tested to generate aggregated results for further evaluation.

#### Step 4: Prompt Evaluation

To systematically assess the performance of various prompt templates developed in steps 2 and 3, a robust sampling strategy is essential for constructing a reliable evaluation dataset tailored to the use case. An expert review panel should be assembled to independently validate the dataset and establish the ground truth. Additionally, a set of evaluation metrics must be carefully selected to ensure alignment with the specific needs of the use case.

##### Sampling Strategy

Given the innate variability of different studies in the solid tumour area, it’s statistically challenging to estimate a true rate of occurrence for late or incorrect detection of Disease Progression in the PD database. We selected all Roche-sponsored phase II and phase III solid tumour studies that utilise Progression-Free Survival (PFS) and/or Objective Response Rate (ORR) as efficacy endpoints, following RECIST 1.1 criteria. Studies were chosen based on strategic importance and enrollment size. Five studies of special interest to the company; with an additional 11 studies selected based on patient enrollment. The evaluation dataset comprised 309 PDs, with approximately 210 randomly selected and 110 containing keywords related to “progression.” The Baseline data composition can be found in Table 5.

**Table 5:** Baseline composition of the sampled PD data

Country	Countunique of Study_Number	Countunique of Deviation_Number
ARGENTINA	1	3
AUSTRALIA	6	16
AUSTRIA	1	2
BELGIUM	3	3
BOSNIA AND HERZEGOVINA	1	1
BRAZIL	5	9
CANADA	5	9
CHILE	4	4
CHINA	4	11
CZECH REPUBLIC	1	1
FRANCE	9	28
GERMANY	6	9
HUNGARY	2	3
INDIA	1	3
ISRAEL	3	5
ITALY	8	27
JAPAN	6	8
KOREA, REPUBLIC OF	4	5
MEXICO	3	4
NETHERLANDS	2	2
NORWAY	1	1
PERU	1	4
POLAND	6	25
RUSSIAN FEDERATION	6	22
SERBIA	1	3
SINGAPORE	2	2
SPAIN	8	31
SWITZERLAND	2	2
TAIWAN	2	2
THAILAND	2	3
TURKIYE	6	13
UKRAINE	4	6
UNITED KINGDOM	4	7
UNITED STATES	8	35
Grand total	11	309

##### Expert Review Panel

Two senior quality leads independently reviewed each PD to determine its relevance to disease progression. Differences were reconciled, and this adjudicated output was treated as ground truth for model evaluation. The baseline composition of Actual Positives (DP and Maybe DP) and Actual Negatives (No DP) is shown in Table 6.

**Table 6:** Expert evaluation results

Actual label expert panel	Countunique of Deviation_Number
DP	44
Maybe DP	60
No DP	205
Grand total	309

##### Prompt Performance

Each prompt version was evaluated using standard metrics, specifically precision and recall.

- *Precision* measures the proportion of correctly identified PDs relevant to patient disease progression among all instances classified as such. It reflects the model’s ability to minimise false positives.
  $${\text{Precision}} = \frac{{{\text{True Positives}}}}{{{\text{True Positives}} + {\text{False Positives}}}}$$
- *Recall* measures the proportion of actual PDs relevant to patient disease progression correctly identified by the model, indicating its ability to minimise false negatives.
  $${\text{Recall}} = \frac{{{\text{True Positives}}}}{{{\text{True Positives}} + {\text{False Negatives}}}}$$

While precision ensures accuracy in identifying relevant records, recall focuses on capturing as many true instances as possible. As outlined in our prompt strategy, we prioritised recall to ensure the model flagged as many relevant PDs as possible, accepting lower precision to minimise the risk of missing relevant cases. We will further elaborate on how we balanced recall and precision through integrated insights and visualizations for expert investigation in section “Balancing Recall and Precision Through Integrated Insights and Visualisations for Experts Review”.

The selected three prompt versions were tested using the evaluation dataset under various combinations of Temperature and Top_P configurations. Zero-shot Version 2 achieved the highest recall across all settings (> 80%), while Version 1 maintained superior precision. Trade-offs between recall and precision were analysed to identify the best-performing configuration. Table 7 summarizes the full metrics, and we will further discuss our findings in the following sections.

**Table 7:** Evaluation results of selected prompt versions

Prompt_version	Temp	Top_P	Precision (%)	Recall (%)
**version_2**	0.8	0.8	49.44	**85.44**
**version_2**	0.5	0.8	49.71	**84.47**
**version_2**	0.5	0.5	50.29	**83.50**
**version_2**	0.2	0.8	49.71	**83.50**
**version_2**	0.8	0.2	49.71	**83.33**
**version_2**	0.2	0.5	49.71	**83.33**
**version_2**	0.5	0.2	49.42	**83.33**
**version_2**	0.2	0.2	49.70	**82.35**
**version_2**	0.8	0.5	49.41	**82.35**
version_3	0.5	0.8	51.77	71.57
version_3	0.2	0.8	50.69	70.87
version_3	0.8	0.5	48.63	70.30
version_3	0.8	0.2	49.66	69.90
version_3	0.2	0.2	48.98	69.90
version_3	0.5	0.2	48.65	69.90
version_3	0.2	0.5	48.97	69.61
version_3	0.5	0.5	49.65	69.31
version_3	0.8	0.8	47.55	68.69
version_1	0.8	0.2	74.47	33.98
version_1	0.8	0.5	73.91	33.01
version_1	0.2	0.5	73.91	33.01
version_1	0.5	0.2	73.91	33.01
version_1	0.2	0.2	70.83	33.01
version_1	0.5	0.5	71.74	32.04
version_1	0.2	0.8	70.21	32.04
version_1	0.5	0.8	72.73	31.07
version_1	0.8	0.8	67.44	28.16

Bold indicates the prompt version that achieved the best performance for our use case under the chosen prompt strategy

##### Prompt Version Analysis

The three prompt versions tested varied in structure and level of contextual detail, as illustrated in Table 4. Version 1 incorporated domain-specific examples, enhancing precision (> 70%), while Version 3 adopted a structured format with examples listed alongside classification criteria but without contextual explanations. The zero-shot Version 2, which omitted examples, achieved the highest recall (> 80%), aligning with our objective and emerged as the best-performing prompt. It included key elements such as the task objective, classification criteria, instructions, and reinforcement (see Table 8). Additionally, strict instructions were implemented to ensure the model focused solely on the provided content, relying on explicit facts while ignoring prior conversations.

**Table 8:** Key elements of prompt version 2

Prompt element	Prompt details
Task objective	As Nova, your task is to identify protocol deviation records related to Disease Progression (DP), also called Progressive Disease (PD), based on RESIST1.1
Criteria for Classification	“DP”: Records related to the assessment or handling of disease progression. Assessment of disease progression includes PI’s disease progression assessment, clinically assessed as disease progression or incorrect assessments “Maybe DP”: Records suggest potential impacts on Disease Progression but lack sufficient detail for a definitive classification “No DP”: Records that are not related to disease progression
Instruction	Review the provided record, applying the specified criteria Respond in JSON format, with “answer” (DP/No DP/Maybe DP) and “explanation” Focus solely on the content of the record provided, basing your decisions on explicit facts from the records without making assumptions. Ignore any prior conversation history and do not generate conversation
Reinforcement	Yes, I understand. I am Nova, and I will classify your records as you instructed. I will respond in JSON format as requested

Version 2 was refined for solid tumour studies using RECIST 1.1 but can be easily adapted for related applications, such as identifying disease progression-relevant data management checks, clinical science reviews, or other PD categories, such as compliance with the informed consent process and dosing regimens. Applying this prompt to other study types or endpoints may require further iteration, following the refinement process outlined in this section.

As the achieved 80% recall was sufficient for this experiment, we prioritised completing the integrated solution (detailed in section “Results”) over further prompt refinement. However, given the rapid advancements in LLMs, future research should explore diverse prompt techniques on larger datasets while leveraging the latest models trained on more comprehensive and up-to-date data to further improve recall and precision. Additionally, evaluating model performance across different PD categories, with a focus on balancing precision, recall, sensitivity, and consistency, could enhance classification accuracy in real-world applications.

## Results

The labelled PDs are further analysed and presented in a visual and interactive dashboard using Tableau®, which enabled dynamic exploration of model predictions and facilitated expert review.

**Figure 3:**
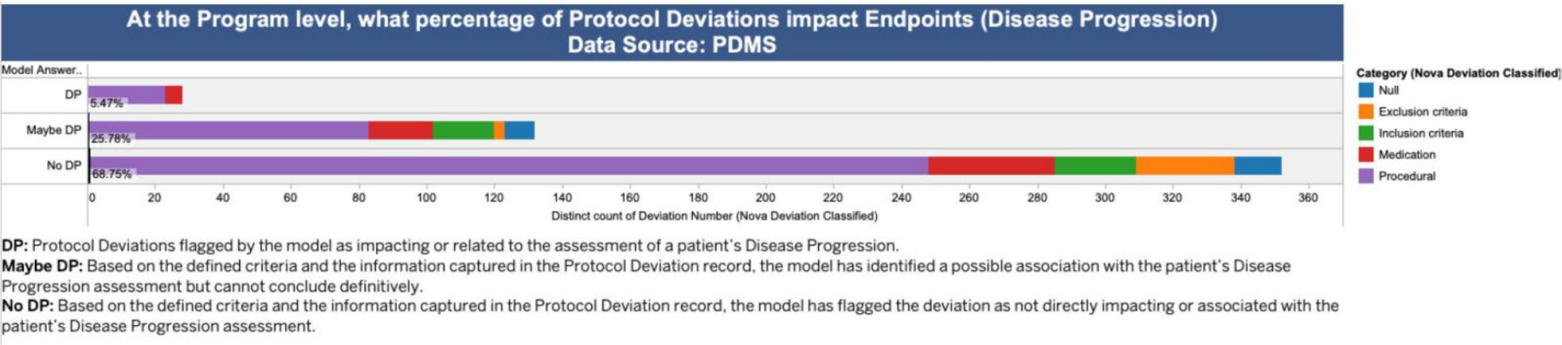
Proportion of PDs that could affect the timeliness and accuracy of disease‑progression handling, grouped by category

Key analytics included:

Figure 3 displays the proportion of PDs that may impact the timeliness and accuracy of handling disease progression in a given program by category (Procedure, Medication, Inclusion, and Exclusion). Users can click on each category to view detailed data, including the model’s label and explanation. This visualisation enables Roche Quality professionals to quickly assess program-level risks associated with disease progression management and identify high-risk areas requiring attention within internal quality controls.

**Figure 4:**
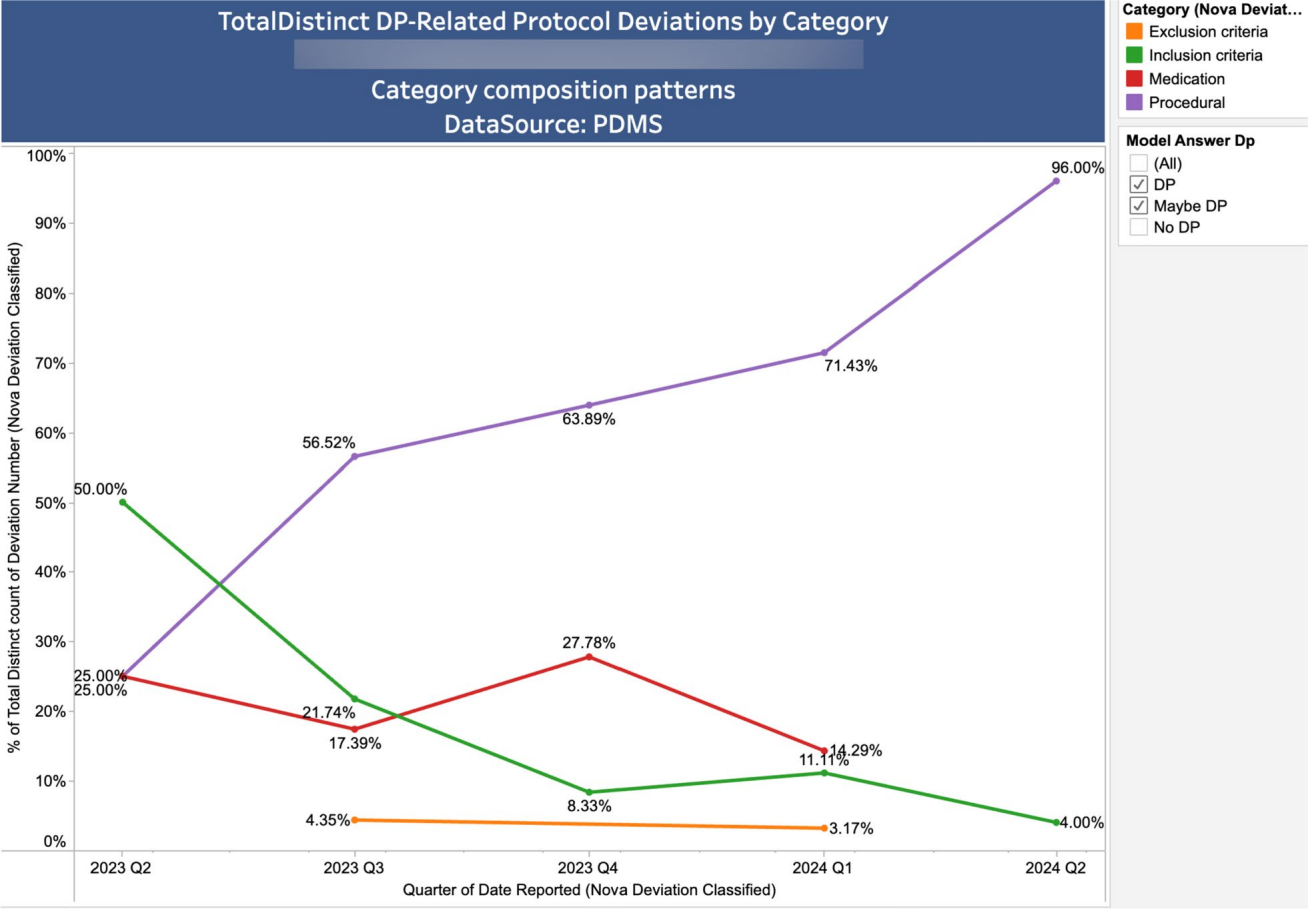
PDs that may impact the timeliness and accuracy of handling disease progression cross relevant studies, trend over time by category

Figure 4 allows the Roche Quality professionals to quickly assess the types of PDs that may impact the timeliness and accuracy of handling disease progression over time across relevant studies.

**Figure 5:**
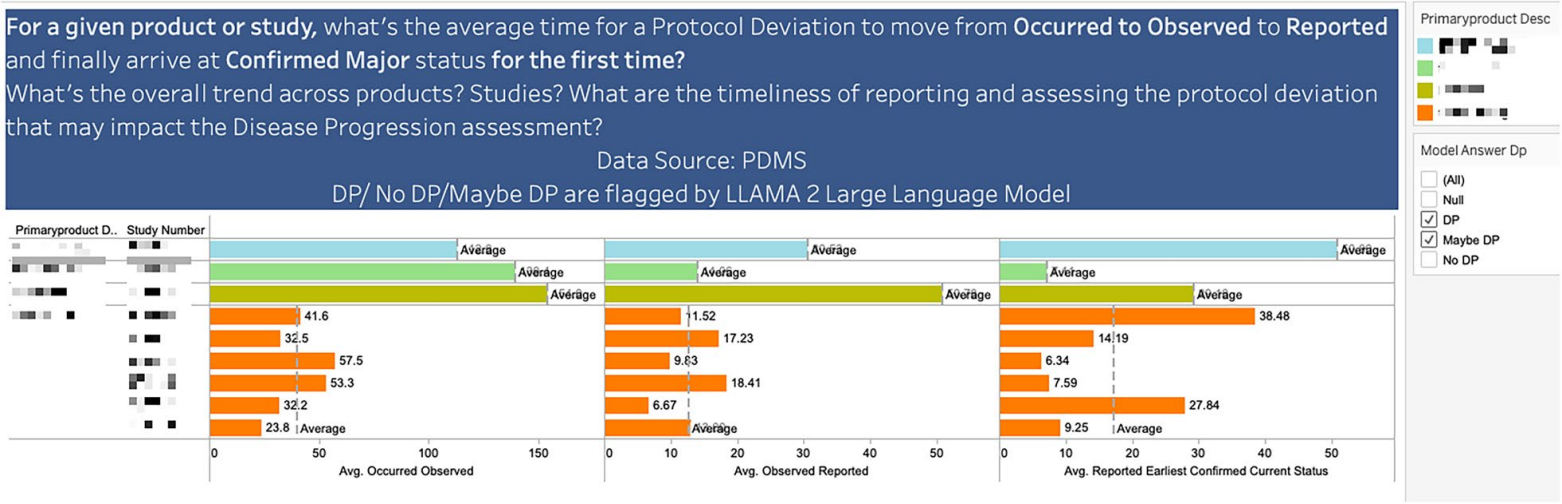
Timeliness in addressing PDs affecting disease‑progression assessment across products, studies, and key process steps

Figure 5 allows the Roche Quality professionals to quickly assess the timeliness of handling such PDs by Product, Study and Key Process Steps.

**Figure 6:**
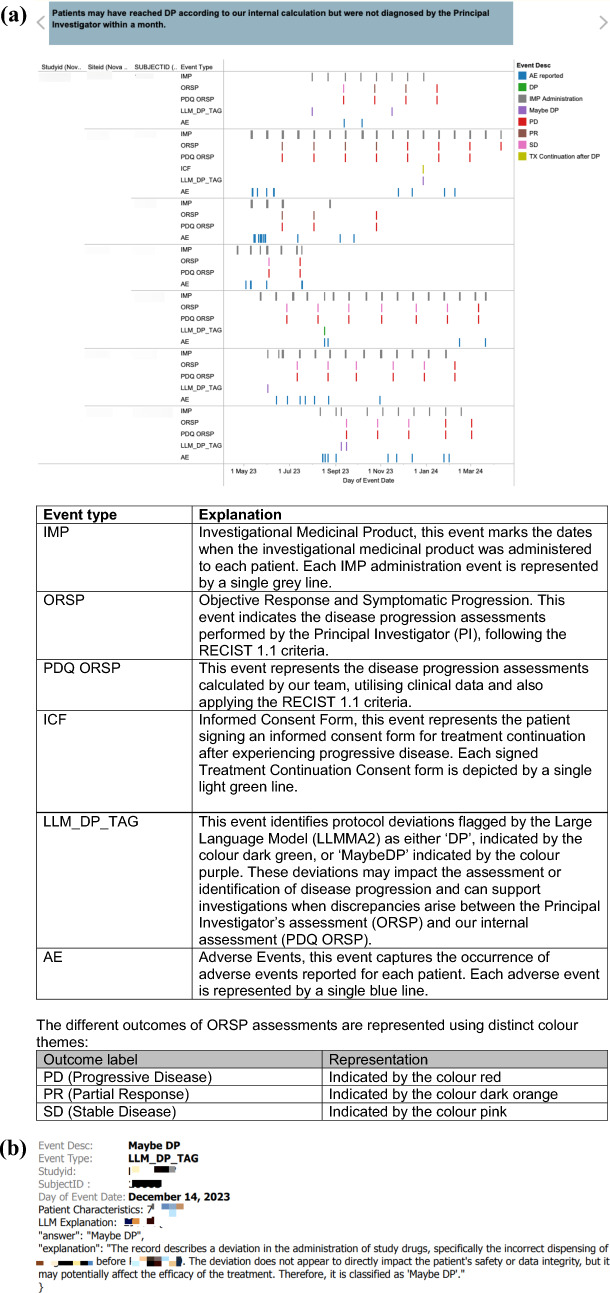
Timelines of key events for patients flagged as progression by internal RECIST 1.1 but assessed differently by the PI within 30 days

Figure 6a presents a timeline visualisation of key clinical events for individual patients identified as potentially reaching disease progression based on our internal RECIST 1.1 calculation but not diagnosed by the Principal Investigator within a month. The data is grouped by Study, Site, Subject, and Event Type, with individual events visualised as single lines, colour-coded by their description and positioned along the timeline of their occurrence date. This visualisation provides experts with a quick, high-level overview of various events that may impact a patient’s journey. By hovering over events, experts can efficiently investigate specific cases and access additional details. Additionally, the model was instructed to generate explanations for its predictions, which are included in the detailed view of LLM_DP_TAG shown in Fig. 6b to support expert assessment and decision-making.

Different Event Types visualised in Fig. 6a are explained in the table below.

## Discussion

### Balancing Recall and Precision Through Integrated Insights and Visualisations for Experts Review

As discussed in the prompt strategy, prioritising recall over precision is a deliberate choice in our use case for several reasons:

- *Low prevalence but high impact*: PDs related to disease progression are infrequent, with only 11% flagged in our evaluation dataset. However, missing these events can have significant consequences.
- *Complex interpretation*: As outlined in section “Step 1: Prompt Strategy Development”, determining whether a PD directly reflects or influences disease progression is inherently complex and varies across experts and study settings. This complexity is reflected in the observed precision rate of approximately 50%.
- *Risk–benefit trade-off*: The impact of failing to identify a true case of disease progression outweighs the burden of reviewing false positives, especially given the substantial manual effort currently required to detect these rare events.

To address the challenges associated with lower precision, the model’s outputs are integrated with relevant clinical data, including disease progression assessments from the principal investigator, internal disease progression calculations, patient consent status for continuing treatment after disease progression, adverse event reports, and dosing information. Additionally, intuitive and contextualised visualisations, as illustrated in Fig. 6a, b, facilitate efficient expert review by presenting relevant events in an accessible format.

Rather than serving as a standalone decision-making system, our solution functions as a decision-support tool, enhancing expert oversight by integrating model’s high-recall outputs with clinical insights. This approach improves the efficiency and comprehensiveness of the review process while minimising the risk of overlooking critical events. Combining automated detection with expert evaluation in a structured, user-centred manner, mitigates the burden of reviewing false positives and addresses the limitations of the current manual, siloed review process outlined in the background section.

### LLMs versus Traditional Methods

As discussed in the background section, a key advantage of using LLMs like Meta Llama 2 lies in their ability to process unstructured text without the need for extensive preprocessing [23], feature engineering and model tuning, steps that make traditional methods more resource-intensive and less flexible.

Our observations highlight the importance of balancing contextual depth in prompts to optimise LLM performance while staying within the constraints of the context window. Providing too much context, as seen in versions 1 and 3, risks exceeding this limit, potentially diminishing performance by restricting the model’s ability to effectively analyse the input.

Moreover, LLMs are not inherently optimised for structured decision-making unless explicitly guided by rules or decision trees. Similar to humans interpreting SOPs and work instructions, LLMs perform best when given clear, straightforward guidance. Traditional rule-based systems and decision trees offer consistency and reliability for tasks with strict criteria. However, they often struggle with unstructured text, as they rely on handcrafted features and rigid rules that may overlook subtle nuances in the language.

In contrast, LLMs are trained on vast and diverse text corpora, enabling them to interpret and generate responses with minimal preprocessing [6]. This capability allows LLMs to handle the nuances and variability inherent in unstructured text more effectively than traditional methods [24]. The high recall score of over 80% achieved in our classification task illustrates the LLM’s ability to identify relevant instances within unstructured text effectively.

In summary, LLMs provide a powerful tool for bridging the gap between unstructured data and structured decision-making. Their ability to process unstructured text with minimal preprocessing offers flexibility and scalability, particularly in contexts where traditional methods struggle with complexity and require extensive manual intervention. This is especially relevant in use cases where high recall is crucial, leveraging the model to sift through vast amounts of unstructured text and identify the majority of relevant cases for further focused review by experts. Striking the right balance between providing enough context and ensuring concise, clear prompts is key to maximising the model’s effectiveness in these applications.

### Proof of Concept (PoC): Key Learnings

This research explores a practical approach leveraging the latest advancements in LLMs, to enhance clinical development. While technology leaders like OpenAI and Meta have made significant progress in improving model performance, the pharmaceutical industry has been slower to adopt, particularly in highly regulated areas such as clinical development and pharmacovigilance. Instead of identifying the best-performing models or prompts, our focus is on key considerations for implementing these technologies and demonstrating their potential value for broader industry adoption.

A key takeaway is the importance of setting realistic expectations and clearly defining the scope of an LLM’s capabilities. As discussed in section “Balancing Recall and Precision Through Integrated Insights and Visualisations for Experts Review”, we applied the model to a labelling task that is both resource-intensive and challenging to perform manually due to the high volume of data and the difficulty in ensuring inter-rater [25] and intra-rater [26] reliability. In our experiment, establishing a baseline ground truth for 309 PDs required two experts approximately 1.5 cumulative working days. The reconciliation process revealed a 20% disagreement between experts and 5–10% intra-rater inconsistency. In contrast, our automated approach processed a single prompt version with one parameter combination in approximately two minutes, yielding a 30% disagreement compared to the ground truth. These findings highlight the advantage of adopting LLMs for labour-intensive text processing tasks.

As discussed in section “LLMs versus Traditional Methods”, LLMs perform best when given clear and straightforward instructions. Identifying PDs affecting disease progression is inherently complex and the choice of this task for our PoC was driven more by its business impact than by technical feasibility. Nevertheless, this classification challenge is representative of broader, often nuanced challenges in clinical development. Our PoC demonstrated that rather than deploying LLMs as stand-alone solutions, their capabilities, such as text mining, might be better suited and more effective when integrated into a comprehensive end-to-end framework, as discussed in section “Balancing Recall and Precision Through Integrated Insights and Visualisations for Experts Review”.

### Practical Challenges for Further Adoption

Our approach, as demonstrated by this successful PoC project, has the potential to be extended to other endpoint types and therapeutic areas, broadening its impact across clinical research. However, scaling this use case for broader adoption presents several practical challenges:

1. The pharmaceutical industry operates within a highly regulated environment, where stringent compliance requirements intersect with human and organisational factors that influence decision-making. Organisations may be risk-averse and may underestimate the multifaceted requirements for meaningful AI integration. Overcoming these challenges requires more than just technological deployment, it demands a holistic approach. Developing high-impact, low-risk use cases is essential to navigate regulatory complexities, as highlighted by Bryce Hall in McKinsey’s 2023 Gen AI survey [27]. This, in turn, calls for strategic frameworks that align human expertise, business needs, and technology, ensuring AI is implemented in a pragmatic and incremental manner that complements human decision-making.
2. While LLMs have generated significant enthusiasm for their ability to process unstructured data and uncover hidden patterns, their real-world effectiveness depends on key factors: Data quality, clearly defined use cases, and seamless integration with domain expertise. Successful AI deployment requires careful calibration, ongoing human oversight, and a robust governance framework to address challenges such as interpretability, regulatory compliance, and bias. Organisations may find it challenging to identify high-impact, practical use cases worth investing in amid the widespread AI-driven marketing narratives, which do not always translate into meaningful value.
3. Deploying AI in a regulated Good x Practice (GxP; e.g., Clinical, Laboratory, Manufacturing, Pharmaceutical) environment raises critical validation questions: Should the solution be validated? To what extent? And how should analytics tools leveraging LLMs for unstructured data processing be validated? Ensuring compliance requires rigorous model validation, maintaining audit trails, and preserving traceability in line with industry regulations. However, existing computer system validation (CSV) frameworks were not originally designed with AI in mind, and regulatory guidelines for AI validation are still evolving. Additionally, given the challenge of establishing a large, validated ground truth dataset from an expert panel, continuous monitoring strategies must be put in place to mitigate potential biases and uphold fairness and ethical standards. Collaboration with cross-company partners, cross-validation using external datasets, and adherence to AI Ethics Principles [28] will be essential to ensuring responsible implementation.

### Limitations

One key limitation of our approach lies in potential biases and subjectivity, as we only analyse the data reported and recorded in the system. This data is heavily influenced by how closely the Roche PD reporting SOPs and guidelines are adhered to across different studies and sites. The lack of detailed descriptions in some PD records may further contribute to variability in interpretation. Reality is inherently complex, and subjectivity is inevitable—there is no perfect model or human expert, making the establishment and maintenance of a robust ground truth baseline a significant challenge. Additionally, prompt sensitivity plays a crucial role in model performance, as higher sensitivity leads to greater variability in output based on input variations. In contrast, consistency measures how uniformly the model performs across similar samples. While high consistency with low sensitivity indicates model stability, future research should evaluate both sensitivity and consistency across larger datasets and multiple models. [20] Finally, it is important to emphases again that the model serves as a technical capability to support decision-making, not to replace human judgment. Roche QA functional responsible remain fully accountable for reviewing protocol deviations and relevant data to ensure patient safety and the integrity of study outcomes.

## Conclusion

In this paper, we proposed a pragmatic approach for leveraging LLMs to automatically label important PDs for further trending and analysis. Our approach provides a practical solution for extracting in-depth insights from unstructured free text in PD records. This method addresses common challenges in PD reporting and trending, enabling efficient, flexible, and targeted PD classification, while complementing the best practices outlined by TransCelerate Protocol Deviations Solutions [29]. Our solution can also be easily adapted for classifying other clinical research data or addressing similar challenges in clinical development. Beyond demonstrating the feasibility of LLM-enabled PD classification, our experiment highlights key considerations for AI adoption in clinical development. Future research should focus on both technical and organisational advancements. Technically, improvements can be achieved through utilising the latest LLM models and refined prompt engineering techniques, and testing on larger datasets. Organisationally, successful adoption will require investment in defining meaningful use cases, establishing clear validation frameworks, and addressing compliance and ethical considerations.

## Data Availability

No datasets were generated or analysed during the current study.
